# Blockchain-enabled secure authentication and privacy-preserving information sharing in VANETs using adaptive echo state networks and dual trapdoor homomorphic encryption

**DOI:** 10.1038/s41598-026-49125-7

**Published:** 2026-06-03

**Authors:** Poonguzhali Ilango, Nagarajan Sivarajan, M. P. Rajakumar, Sumanth Venugopal

**Affiliations:** 1 Department of Electronics and Communication Engineering, Panimalar Engineering College, Chennai, Tamil Nadu 600123 India; 2 Department of Electronics and Communication Engineering, SRM Institute of Science and Technology, Ramapuram, Chennai, Tamil Nadu 600089 India; 3 Department of Artificial Intelligence and Data Science, St.Joseph’s College of Engineering, Old Mamallapuram Road, Chennai, Tamil Nadu 600 119 India; 4 Manipal Institute of Technology Bengaluru, Manipal Academy of Higher Education, Manipal, India

**Keywords:** Adaptive blockchain-based authentication procedure, VANET, Adaptive echo state network, Position updated enhanced frilled lizard optimization, Dual trapdoor homomorphic encryption with optimal multi-key, Engineering, Mathematics and computing

## Abstract

A blockchain-driven approach is presented to ensure robust authentication and private data distribution in Vehicular Ad Hoc Networks (VANETs). A primary objective to develop a robust authentication procedure using an Adaptive Echo State Network (AESNet) and a dual trapdoor homomorphic encryption method (DTHE-OMK) to enhance secure data transmission in VANETs. This work employs a combination of optimization strategies, including the Position Updated Enhanced Frilled Lizard Optimization (PUE-FLO) algorithm, to fine-tune the parameters of the AESNet for node authentication. The authentication analysis on accuracy provided results in the range of 6.323% of CWO-AESNet, 5.828% of TOT-AESNet, 8.353% of WOA-AESNet, 6.323% of FLO-AESNet for the optimization algorithms and 7.838% of DNN, 4.729% of LSTM, 4.248% of GRU, and 4.729% of ESNet for various literature models, respectively. This work concludes that the developed approach improves both integrity and safety of information shared across VANETs.

## Introduction

The rapid advancement of Information Technology, along with VANET architectures, has captured significant interest in enhancing road traffic control and safety^1^. The operation of VANETs typically executes through three core modes of communication^2^. These communication methods rely on dedicated short-range wireless connectivity within vehicular environments to enable efficient data exchange among network entities^3^**.** Despite progress, several persistent challenges remain, including security vulnerabilities, limited scalability, lack of flexibility, and issues with system configurations^4^. To address these concerns, consortium blockchain technology has been adopted in recent studies related to vehicular management systems, thereby promoting transparency and maintaining immutable records of vehicular activities^5^.The cryptographic capabilities of these systems help mitigate risks such as transmission snooping and data interruption^6^. However, blockchain implementation introduces significant computational overhead, which can be alleviated through the integration of 6G networks and edge cloud computing infrastructure^7^. Furthermore, a decentralized blockchain-based data integrity audit mechanism has been proposed to enhance both security and operational efficiency in VANET environments.

Despite these advancements, persistent challenges remain, such as limited coverage, high communication costs, and delays in traffic incident notifications^8^. While IoT devices support efficient vehicle communication^9^, concerns regarding connectivity, response speed, and service scalability continue to hinder overall system performance^10^. Software Defined Networking for Vehicles (SDNV) generates data that is stored within cloud infrastructure, enabling on-demand scalability and facilitating further processing, thereby playing a pivotal role in providing ubiquitous network access for a wide range of applications^11^. However, these services typically operate with minimal user control and limited interaction with service providers. This dependency may lead to increased latency, especially in emergency situations such as vehicle theft or accidents**,** where rapid response is critical^12^.Another major concern within VANETs is fault tolerance. In scenarios where crucial or emergency functions fail, passengers and tourists may remain unaware of service disruptions due to inadequate verification protocols at local servers^13^.When a vehicle communicates with nearby edge servers, uncertainty often arises as to whether its message is received by a legitimate server or intercepted by a malicious entity**.** As a result, ensuring secure message delivery with minimal communication delay remains a central challenge for VANETs^14^.

Sophisticated neural networks demonstrated exceptional capability in evaluating and pinpointing safety risks within VANETs. The effectiveness of VANET security mechanisms largely depends on the adaptability of algorithms to the dynamic nature of vehicular systems^15^. In large-scale wireless environments, deep learning-based safety solutions enable early detection of cyber-attacks with a commendable level of accuracy and timely response^16^. To safeguard distributed network architectures against sophisticated cyber threats and to enhance resilience, blockchain technology has been employed to address critical security and privacy concerns^17^. Numerous methods for spotting threats was integrated into vehicle networks for enhancing overall defense and achieve faster response times. Additionally, an advanced and user-centric VANET model has been introduced to deliver services such as secure navigation, intelligent mobility, traffic congestion control, entertainment, and overall safety, making it a promising solution for modern vehicular networks. However**,** to address current challenges and the complexity of existing authentication mechanisms a blockchain-driven system for secure authentication and communication within VANETs is introduced, aiming to simplify complex security protocols while improving the system’s flexibility and operational efficiency.

- *Development of blockchain-based authentication*: To engineer a novel deep learaning architecture that enables secure data transmission with an improved authentication process in the VANET architecture. The necessity of authentication is vital in data sharing for blockchain-based VANETs to ensure the trustworthiness and security of shared information. This authentication aspect of the system verifies the identity of participating vehicles and entities, preventing unauthorized access and malicious data manipulation. Therefore, VANETs can effectively communicate and share data with robust authentication mechanisms, which are critical for maintaining network integrity and safety.
- *Development of node authentication framework*: To leverage node authentication via blockchain for facilitating secure distribution of data among connected VANETs. The developed AESNet network offers secure data transmission in VANETs that ensures verification of every blockchain node**,** regardless of whether it is authenticated or not. The application of the Echo State Network (ESNet) provides enhanced system authentication that iteratively analyzes the complex patterns in vehicle communication data and improves secure data transfer in VANETs. This allows for reliable data sharing without any security breaches. This intervention strengthens the node authentication and access decisions of the blockchain with improved accuracy. Thus, critical data transfer is accomplished securely in VANETs without any malicious interference.
- *Parameter tuning using improved algorithm*: To implement an innovative PUE-FLO heuristic algorithm for optimal parameter tuning. The optimal parameter tuning improves the effectiveness of authenticating the nodes. Performance of the AESNet-based identity check was improved by refining the network’s activation methods and internal processing units such as number of hidden units, and system learning rate. The main intention of applying the developed PUE-FLO heuristic algorithm to the AESNet network is to achieve higher accuracy in node authentication. The higher accuracy factor indicates the selection of authenticated nodes to store and share data in the VANET environment. Hence, the secrecy of the data transmission is ensured in the blockchain-stored data.
- *Multi key encryption process*: To introduce an innovative cryptographic algorithm and advanced encryption and decryption method, DTHE-OMK for safe vehicular communication. Its optimized multi-key architecture allows for secure, decentralized computation, where every node manages its own homomorphically-generated credentials, which allows encrypted data from different sources to be jointly processed, and the dual trapdoor structure facilitates seamless key switching to ensure compatibility across multiple keys. This key generation is optimally fine-tuned with the developed PUE-FLO optimization scheme, which dynamically tunes key parameters such as binary-formatted public and private keys. As a result, the DTHE-OMK cryptography tool maintains robust privacy guarantees, thus making it shows potential for security-sensitive scenarios.

*Organization of the research manuscript*: Here, the structure of the research work is explored in detail. Section "Literature survey" evaluates foundational literature and related academic contributions. Section "Development of blockchain-based secured authentication and information-sharing model in VANET and its network architecture with the significance" discusses design and significance of a blockchain-based secure authentication and information-sharing model in VANET, along with its network architecture. Section "Node authentication and access decision using the proposed adaptive network with enhanced position updated optimization algorithm" explains the proposed adaptive network’s node authentication and access control mechanism, using an improved position update optimization algorithm. Section "Encryption and decryption for information-sharing using the developed optimal multikey generation-based cryptography technique" introduces an optimal multi-key generation cryptography technique for secure data sharing. Section "Results and discussion" presents numerical outcomes and performance discussion, while Sect. "Conclusion" provides final deductions of this research.

## Literature survey

In 2022, Ahmed et al.^18^ recommended a Software-defined Fault Tolerance and Quality of Service-Aware (DFT-QoS) system that utilizes secure blockchain technology to enhance communication in VANETs**.** It addressed various challenges in VANET-based data transmission using blockchain and improved system efficiency. By integrating fault tolerance mechanisms, it helped ensure smoother and safer data exchanges between vehicles. A data indicates 55% reduction in total message propagation time for all traffic types, achieved through deployment of edge-based SDN management within proposed architecture.

In 2022, Zhang et al.^19^ developed a BMEC-FV framework that introduced a blockchain-based hierarchical Multi-Access Edge Computing (MEC) model for the future VANET ecosystem. This research focused on maximizing data rates and service quality for edge-computing participants at foundational level of network’s design. Furthermore, it employed a trust model for V2V communication. Since traditional approaches were ineffective, an innovative deep compressed neural network was developed and evaluated. The framework improved data handling and communication security within VANETs.

In 2024, Mohammed et al.^20^ suggested a re-authentication scheme called the Handover Authentication Scheme Based on Fog Computing (HAFC), which enables rapid re-authorization of vehicles through secure asset transfer. The HAFC scheme was consisted of two primary stages including, initial authentication and handover authentication. Based on experimental analysis, the handover mechanism-based authentication stage has achieved a minimal processing time than the initial authentication stage.

In 2022**,** Chen et al.^21^ recommended a Decentralized VANET (DVANET) model in which computational procedures were decomposed from centralized cloud services to Edge Computing (EC) nodes**.** This minimized system delays and other disadvantages of conventional approaches in secure data transfer. To ensure protected data exchange among mobile nodes and roadside points, this study has leveraged a immutable ledger to manage a Decentralized Privacy-preserving Deep Learning (DPDL) update cycle, enabling the transparent storage and sharing of local model parameters without central authority.

In 2025, Kalidoss et al.^22^ introduced a deep learning model integrated with blockchain to enhance VANET security**.** The network configuration comprised three layers. This underwent rigorous testing with comprehensive data provided to validate its performance. The results demonstrated strong performance in protecting data exchanged between vehicles. This model focused on verifying integrity of blockchain-managed vehicle nodes and employed an Adaptive Dilated Gated Recurrent Unit (AD-GRU) for node authentication, utilizing an Enhanced Osprey Optimization Algorithm (EOOA) to achieve superior parameter calibration.

In 2022, Poongodi et al.^23^ suggested an effective batch authentication and key exchange technique to prevent interactions with hostile vehicle users in the VANET architecture. To manage conflict between ledger synchronization and network speed, this work has utilized a Markov optimization model. The proposed framework was organized into perception, edge, and service stages where each level contributes to safety of VANET communications. Specifically, the architecture has secured raw data at point of entry and utilized a dual-storage strategy at the service level to maintain system-wide trust.

In 2024, Razaque et al.^24^ discussed how wireless capabilities could be improved using a 6G network to manage IoT devices in Vehicular Communication Systems (VCSs). The study introduced an energy-conscious 6G network backed by blockchain to facilitate safe vehicular management. This hybrid model has combined edge-cloud processing and blockchain to deliver rapid response capabilities and lower electricity consumption. Its primary intent was to inspire researchers to explore integrated multi-disciplinary approaches for network design.

In 2024, Minu et al.^25^ established an innovative approach to address the limitations of conventional models by enhancing privacy and security protocols through a fog computing-based VANET architecture. The suggested architecture centers on three core pillars identity validation, data confidentiality, and message integrity. To improve authorization success rate of vehicle nodes, an Adaptive Deep Bayesian Network (ADBN) was employed, with its internal variables calibrated via a combined Fire Hawk and Tunicate Swarm (IFHTSA) optimization. For safeguarding sensitive information, a Hybrid Attribute-Based AES (HABAES) approach was implemented, yielding 94% accuracy and 93% precision.

In 2023, Bala et al*.*^26^ developed a Blockchain-enabled Trust and Location-dependent Privacy-Preserving (BTLB-PP) authentication system to address security and tracking vulnerabilities in Intelligent Transportation Systems (ITS) and VANETs. The study has implemented Dirichlet distribution trust management model to facilitate collaboration only with trusted vehicles using blockchain to securely register trust scores and location data. When evaluated through simulated traffic scenarios, the BTLB-PP system have demonstrated enhanced resilience against trust model for achieving high efficiency and practical security.

In 2025, Liu et al*.*^27^ proposed a Lightweight Authentication and Privacy-Preserving Aggregation Scheme for Blockchain-Enabled Federated Learning (LPBFL) in VANETs. The authors have constructed a distributed secure authentication framework to enable efficient vehicle validation and secure transmission of models. They further implemented simplified three-party key agreement utilizing chaotic maps to generate session keys, ensuring private transfer of intermediate models. Both security analysis and formal verification confirm that LPBFL bolsters reliability and privacy of underlying model data.

In 2026, Bhatt and Kar^28^ proposed a novel blockchain-based authentication scheme that leveraged conditional privacy within temporary platoons. This extended model was leveraged based on temporary blockchain platoons formation, which utilized blockchain technology to ensure data integrity and prevent unauthorized access while limiting data sharing to pre-defined trust levels. By significantly reducing reliance on Roadside Units (RSUs), the scheme provided a cost-effective traditional public key infrastructure to promote a wider VANET implementation. Furthermore, the authors have incorporated an accident prevention mechanism for improving system-wide reliability and safety, while maintaining core mechanisms of the original platoon model.

In 2024, Rawat et al*.*^29^ proposed Blockchain-assisted Trust Computation with a Conditional Privacy-preserving Authentication (BTC2PA) scheme to the connected vehicles. This scheme has utilized an Ethereum-assisted PKI and digital signatures to achieve robust security for all network communications. Researchers have validated the feasibility of the BTC2PA scheme through implementation on the Rinkeby test network and extensive simulations using NS-3. Findings indicate developed framework notably optimized operational efficiency, specifically reducing transmission overhead, memory requirements, and processing expenses, while also minimizing delivery latency relative to existing solutions.

In 2025 Srivastava et al*.*^30^ addressed significant vulnerabilities in centralized architectures such as high latency, single points of failure, and susceptibility to data breaches. To overcome these limitations, the authors have proposed unique decentralized blockchain framework for identifying verification within connected and self-driving car environments. This methodology has utilized distributed digital IDs and validated certificates to ensure protected authorization of automobiles across a non-centralized network. Comprehensive empirical testing have demonstrated a significant improvement in latency, trust, and resilience to attacks for validating effectiveness of the method compared to conventional systems.

In 2025, Chaurasia et al*.*^31^ presented a blockchain-based key management scheme for the social Internet of Vehicles-Networks (IoV-N). This work has utilized Non-Fungible Tokens (NFTs) and InterPlanetary File System (IPFS) to store records such as cryptographic keys and issuer identities on behalf of trusted authorities. The authors have introduced bilinear pairing for secure key generation and shifting the key generation process to the fog layer to reduce latency while maintaining the blockchain on cloud-based data servers. Extensive simulations have demonstrated that this approach provided a secure, fast, and viable solution for key management in decentralized vehicular networks.

### Problem statement

Presently, the VANET technology is receiving a huge amount of attention from users as they are connected with the vehicular communication models. Maintaining the authentication as well as privacy in VANET is complicated. Broadcasting fake news on the network leads to more vulnerability, and also, security in the decentralized network is affected. Thus, maintaining security and authentication in VANET remains an open challenge. Different issues that arise while designing innovative authentication and information sharing models are detailed as follows.

- The VANET models are prone to scalability issues while handling enormous data in the data transaction phase. Moreover, it needs to maintain the latency, storage and power.
- Maintaining the authentication of the user is complicated as attackers try to misuse the sensitive information.
- Trust management in the network is complicated due to malicious activities of the vehicle and false information.
- In most cases, the VANET models are prone to several attacks, which affect the privacy and security.
- Managing the interoperability among blockchain is challenging as the blockchain model lacks reliability and faces more attacks.
- Improving access control and managing the keys helps to maintain the security of the user information.

On considering these research gaps, a secure blockchain-based authentication and information sharing scheme for VANET was implanted. A comprehensive summary of evolving progress and technical hurdles associated with conventional verification and sharing protocols is detailed on Table 1.

**Table 1:** Specific functionalities and possible drawbacks in classical blockchain-based authentication and information sharing scheme for VANET.

Author [citation]	Procedural approach	System characteristics	Limitations
Ahmed et al*.*^18^	FTQA-SSDVN	• This effectively eliminates communication delay that arises in VANET • Its reliability is higher, and it also minimizes downtime issues	• The intricate architecture results in substantial processing latency • The implementation process is expensive
Zhang et al*.*^19^	BMEC-FV	• It effectively ensures the security of the communication link and vehicle • It is capable of computing the trust among the vehicles through the offloading process	• This is essential to resolve propagation lag and manage high-volume traffic effectively • It requires overcoming the authentication overhead issues
Mohammed et al*.*^20^	CDH	• It maintains the security of keys while transmitting the information in a complex environment • It maintains higher security while sharing the sensitive information	• It needs to maintain the integrity and confidentiality • It requires reducing the processing power and addressing the delay issues
Chen et al*.*^21^	FHE	• It easily tackles issues like congestion, delay, and communication overhead • The model improves collective reliability and protection of sensitive information	• The system necessitates addressing processing overhead to ensure operational efficiency • Storage-related issues need to be tackled
Kalidoss*et al*^22^	AD-GRU	• It tackles the vanishing gradient issues • It is capable of sourcing the temporal and spatial features	• It needs to tackle the validation complication and also requires improving the performance
Poongodi*et al*^23^	HDTMC	• It easily learns the features from longer sequences • The system combines procedural simplicity with robust ability to alleviate network lag	• It is essential to mitigate algorithmic overhead associated with existing processes • The system necessitates enhanced architectural adaptability to handle diverse network conditions
Razaque*et al*^24^	EBH	• The trust and security was improved • This approach bolsters accountability across network and maintains higher standard of information accuracy	• Enhancing model transparency and explainability remains critical requirement for this framework • Its implementation is expensive and needs to consider the privacy concerns
Minu*et al.*^25^	HABAES	• It offers higher security and also maintains overall robustness • It improves the scalability and also needs minimal storage space	• The system requires streamlining verification process to minimize computational strain • Managing keys is complicated

## Development of blockchain-based secured authentication and information-sharing model in VANET and its network architecture with the significance

### VANET: network architecture

**Fig. 1:**
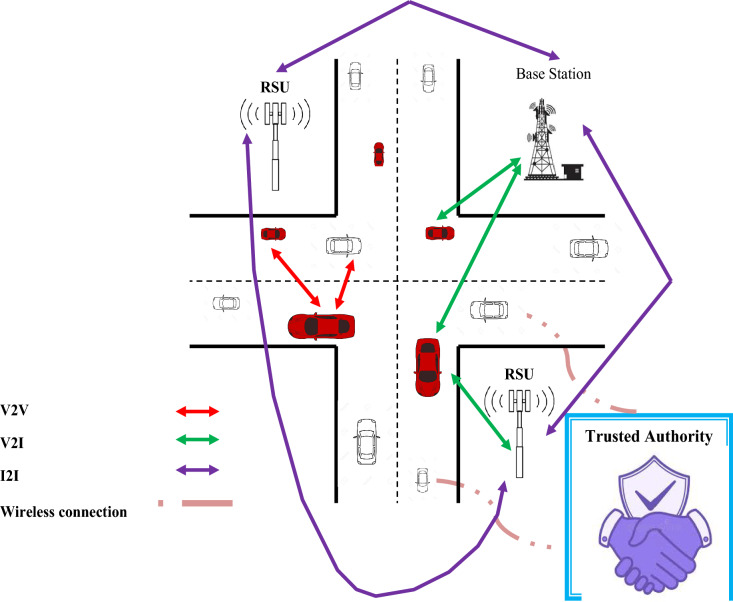
Structural overview of VANET architecture.

As a modern extension of mobile network architecture, VANET enables seamless connectivity for vehicles. This allows for rapid exchange of critical telemetry, including location data, hazard alerts, and real-time road conditions, among all connected entities. This can help drivers stay safe and improve traffic flow without congestion. In this system, the user could receive instant alerts about accidents, road closures, or sudden braking events from nearby vehicles, which offers virtual support for the user’s journey. Moreover, VANETs rely on a special type of communication called Dedicated Short-Range Communication (DSRC), which allows vehicles for propagating and processing wirelessly within the management system. In general, VANET technology transforms vehicles into intelligently connected devices that make roads safer, traffic more efficient and driving more secure. The diagrammatic overview of the VANET architecture was shown at Fig. 1.

### Significance of blockchain infused with a deep learning model

The popularity of the Blockchain has evolved into a decentralized architecture that enhances data security and transparency by eliminating the vulnerabilities of hub-based centralized systems. The method was highly suited for MEC settings, allowing users only rely on situational and spatial awareness. However, it also faces secrecy risks when transferring sensitive data to third parties. Therefore, the Blockchain offers solutions to these problematic scenarios by safeguarding system pathways, user and equipment authorization, and condition vulnerabilities from potential threats. The reach of distributed ledger technology extends far beyond banking, playing crucial role in modern healthcare systems, global supply chains, and secure identity protocols. Despite its transformative effectiveness, blockchain faces significant obstacles, including scalability, technical constraints, and unclear regulations. Therefore, deep learning infused with blockchain-based data transfer gains importance in the application of VANETS to train and handle huge data with limited system intractability.

### Descriptive depiction of the developed blockchain-based deep learning framework in VANET

Blockchain^32^ is more widely acknowledged for its ability to create, secure, and decentralize the operations of systems across various industries. However, its integration faces complications such as technical complexity and regulatory ambiguity. To tackle these issues, a blockchain-based VANET security architecture has been proposed in the current research work. This design is structured in three layers, which comprise vehicles and Roadside Units (RSUs) forming the base blockchain network, supported by edge processing MEC networks for accomplishing computational tasks with node authentication and cryptography-protocol-based blockchain storage^33^. The operations of RSUs use blockchain to oversee and maintain records even while undergoing vehicular movement. To avoid network delays, which are foreseen potential complications in VANET architectures, resource-heavy processes such as image analysis are offloaded to MEC, which returns the results to RSUs. The power requirement of the blockchain is not only associated with the storage of critical data, such as accident reports, but also depends on infractions, such as violations of security protocols. The node authentication for the developed deep learning-based secure blockchain-based authentication in VANET is achieved via the implemented AESNet with optimally tuned hyperparameters using PUE-FLO. Although VANET data is often stored in central systems, the proposed model leverages cryptography protocols while preserving privacy through blockchain with the application of the developed DTHE-OMK strategy.

**Fig. 2:**
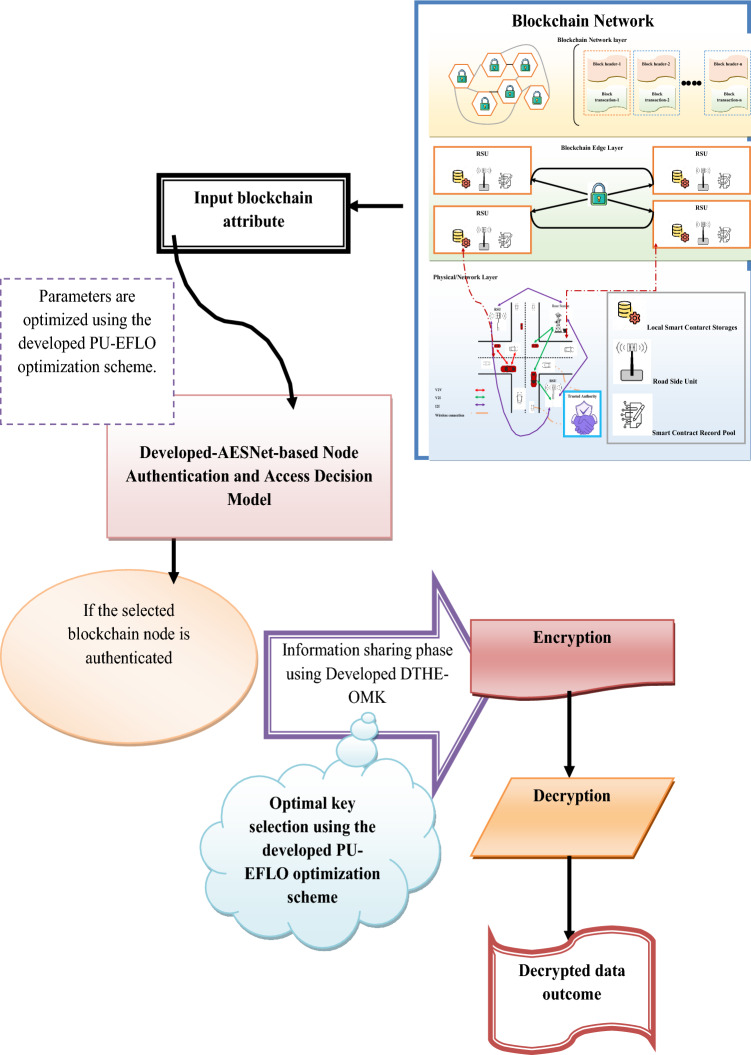
Systematic visualization of developed framework in VANET.

The application of the DTHE-OMK optimizes the VANET-based input attributes and generates a multi-key. This cryptographically design promotes efficiency, scalability, and robust data security in VANET environments. Thus, the developed deep learning and secured blockchain-based authentication and data transfer procedure in the VANET will enhance the system’s resistance to intrusions with the application of elevated cryptographic algorithms. Figure 2 depicts the systematic visualization of the developed Blockchain-based deep learning framework in VANET. The raw vehicular data, like speed, sensor, reading and location, are the input blockchain attributes collected and are securely stored across the network. The developed DTHE-OMK is used for the information sharing process here the DTHE-OMK is used for encrypting the vehicular data. Without exposing the data, the computations are allowed through this encryption process. The DTHE-OMK module is used for the encryption of the collected data. In order to perform the collaborative processing, secure multi-keys are developed in this model. The AESNet is used for authenticating the nodes before the sharing process. In the data exchange process, only legitimate nodes participate because of this operation. Within the blockchain layered architecture, the authenticated records and encrypted data are stored. The keys generated in the encryption process are used for decrypting. Here, the integrity and the privacy of the information are preserved through this model, and it also enables secure information sharing. During the computations necessary for the VANET operation, confidentiality is maintained through this model since it can support the processing of the encrypted data directly.

#### Technical description of blockchain-enabled secure authentication

In this research study, blockchain-enabled secure authentication is a multi-layered technical process designed to ensure integrity, and trustworthiness of data sharing in VANETs. The blockchain-based authentication architecture strictly verifies the identity of every vehicle and entity in the network. This non-centralized record acts as resistant audit trail, preventing fraudulent access and protecting against corrupt data modifications. By distributing the authentication process across the network, the system eliminates single points of failure and serves as dependable basis for verified connectivity and safe distribution of essential road-safety data. The framework further strengthens security through a specialized node authentication framework known as AESNet network. This system iteratively analyzes complex communication patterns within vehicular data to distinguish between authenticated and unauthenticated blockchain nodes. To maximize the precision of these access decisions, the PUE-FLO heuristic algorithm is implemented for optimal parameter tuning. This algorithm refines critical system parameters such as learning rates and hidden units to achieve high-accuracy node selection. By ensuring that only verified nodes are permitted to store and share data, the system effectively maintains security of information stored within the blockchain. Finally, the technical architecture incorporates the DTHE-OMK cryptographic algorithm to facilitate secure collaborative computation. This mechanism utilizes an optimal multi-key generation process where each node independently produces unique public–private key pairs using dual-trapdoor homomorphic structure. This allows encrypted data from multiple sources to be processed jointly without revealing the underlying information. These keys are dynamically fine-tuned by PUE-FLO scheme to ensure compatibility and robustness across decentralized network. Consequently, the integration of deep learning, heuristic optimization, and advanced homomorphic encryption creates an end-to-end secure environment that is highly resilient to privacy breaches and external interference. The blockchain used for authentication is displayed in Table 2.

**Table 2:** Specific blockchain components.

Component	Used approach
Blockchain type	Consortium (permissioned)
Consensus	PBFT
Authentication	AESNet (deep learning)
Encryption	DTHE-OMK (homomorphic encryption)
Participants	Vehicles + RSUs + edge nodes

## Node authentication and access decision using the proposed adaptive network with enhanced position updated optimization algorithm

### Development of PUE-FLO

The suggested PUE-FLO metaheuristic method is an impactful choice for the improvement of secure authentication and data transfer in the developed deep learning and blockchain-based VANET systems. By imitating the adaptive defence mechanisms that are inbuilt in the PUE-FLO optimization strategy, the dynamicity of the system is enhanced for rapid data exchange, real-time decision-making and secure authentication among high-speed vehicle nodes. The implemented PUE-FLO strategy aids in optimizing vehicle positioning and clustering strategies for reliable network topology and efficient resource allocation.

The developed PUE-FLO metaheuristic method is driven by the classical Frilled Lizard Optimization (FLO)^34^. The movement strategies of the frill-necked lizard species are the backbone of this strategy that enhances convergence speed and solution diversity in dynamic environments. This optimization technique is critical for fine-tuning the node behaviour, mitigating congestion, and reducing the system latency during authentication procedures. The position updating criteria of the conventional FLO optimization strategy are presented in Eq. (1).

1 $$F_{a,p}^{C1} = F_{a,p} + WR_{nd}^{NB} .\,\left( {UB_{a,p} - A.F_{a,p} } \right).$$

In this context, $WR_{nd}^{NB}$ is random value within a range of $\left[ {0,1} \right]$, where randomly chosen focal point denotes *A* in solution space of dimension $F_{a,p}$, $UB_{a,p}$ is the solution space boundaries regarding the dimensions *a* and *p*. The random value that existed in the classical FLO heuristic strategy fails to accurately locate the base member in the holistic population, which can lead to suboptimal solutions, slower convergence, and potential vulnerabilities while analyzing the complex and dynamic environment of blockchain. To enhance randomized update phase within conventional FLO framework, the adaptive PUE-FLO approach was introduced with its mathematical formulation provided in Eq. (2).

#### Novelty

The PUE-FLO metaheuristic framework enhances blockchain-driven authentication in VANETs by optimizing AESNet network parameters like hidden nodes, learning rates, and activation functions. By utilizing adaptive mechanism in Eq. (2) to refine random value in Eq. (1), this approach effectively manages high-speed vehicular authentication demands while reducing congestion and latency. In order to handle the inherent unpredictability and temporal variability of VANETs, where network topology, node density, and interaction reliability change quickly over time, a robust adaptive component was modified in the exploration phase of the PUE-FLO. The modifications in the adaptive component of the algorithm prevent the trapping in the condition of the local optimal and it facilitate the discovery of global optimum. Since reservoir dynamics in traditional ESN models are fixed after initialization, the authentication process is vulnerable to sudden behavioural shifts brought on by malicious node activity, channel interference, or mobility patterns. When the statistical distribution of vehicle communication data changes during runtime this restriction frequently results in worse classification performance.

2 $$F_{a,p}^{C1} = F_{a,p} + \frac{{\left( {GC_{ft} } \right)^{2} }}{{\left( {GW_{ft} } \right)^{2} + GB_{ft} }}.\,\left( {UB_{a,p} - A.F_{a,p} } \right).$$

Here, the terms $GC_{ft}$,$GB_{ft}$ and $GW_{ft}$ are current fitness, best fitness and worst fitness, respectively, for the random value $WR_{nd}^{NB}$ within the range of $\left[ {0,1} \right]$ where *A* is the randomly chosen focal point in the solution space of the dimension $F_{a,p}$. Furthermore, the importance of the developed PUE-FLO strategy complements the implemented AESNet network by improving the system performance with optimal parameter tuning, which results in accurate trust score generation for securing the data transfers in the VANETs.

**Fig. 3:**
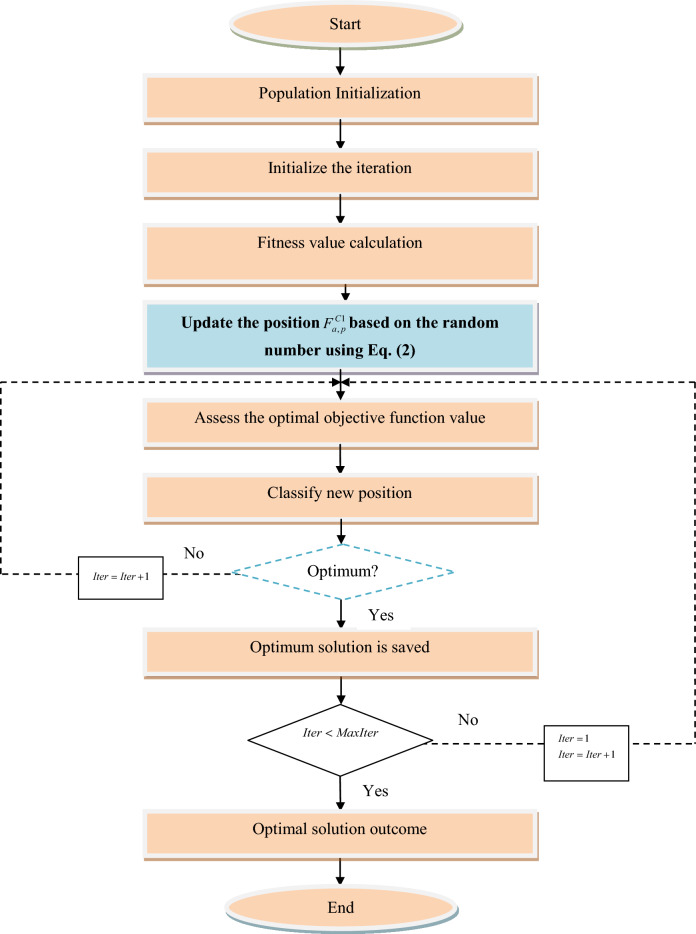
Flow chart of implemented algorithm.

The combination of blockchain in a developed secure data transfer model enhances optimization, ensuring secure and tamper-proof data management across decentralized vehicular nodes. The adaptive positioning of the PUE-FLO heuristic strategy directly influences the path selection and load balancing, thereby preserving bandwidth and improving end-to-end security in the VANETs. Therefore, the deployment of the developed PUE-FLO heuristic approach adds robust intelligence and scalability to the VANET ecosystem, making authentication faster, more reliable, and resilient against evolving threats in intelligent transportation infrastructures. The pseudocode and flowchart of the implemented PUE-FLO heuristic scheme is presented in Algorithm 1 and Fig. 3.

**Algorithm 1:**
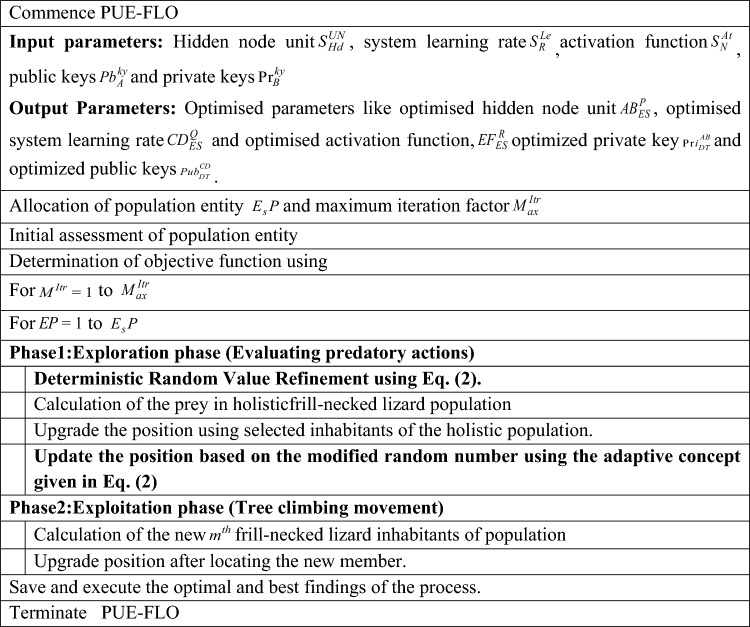
Implemented PUE-FLO algorithm.

### Echo state networks

The Echo State Network (ESNet)^35^ is a unique category of Recurrent Neural Network (RNN) that is known for its effectiveness in processing sequential data. This network depends on pre-defined, randomly linked reservoir to provide short-term memory of past input sequences. Unlike typical feed-forward neural networks, the ESNet requires that the output layer be well-trained to make the learning rate faster and more stable. The reservoir nature of the ESNet, evolving internal states like echoes bouncing around, helps capture patterns and temporal relationships in data, making ESNet particularly useful in time-series prediction and wide applications related to it. The reservoir unit represented as *P* input reservoir, *Q* neuron units, and *R* output unit of ESNet. The processing dynamics of matrix weights such as input, reservoir, feedback, and output can be presented as $G_{I}$,*G*,$G_{B}$ and $G_{O}$. The size of the matrices exists as the combination of the neurons such as *P × Q*,*P × P*,*P × R* and $R \times \left( {P + Q} \right)$. The operations of the ESNet can be defined as follows in Eq. (3) and (4).

3 $$T_{r} \left( {x + 1} \right) = h\left( {G_{I} e\left( {x + 1} \right) + GT_{r} \left( x \right) + G_{B} d\left( x \right)} \right),$$

4 $$u\left( {x + 1} \right) = g^{O} \left( {H_{O} T_{r} \left( {x + 1} \right)} \right).$$

Here, the term *g* is sigmoid activation function and $g^{O}$ is output function for external input state $e = e\left( x \right)$, reservoir state $s = s\left( x \right)$, and output state $u = u\left( x \right)$, respectively. These factors are learned by linear regression. To perform kernel-based learning algorithms, the analysis of available temporal information in the inputs is used. As a result, ESNet remains highly scalable solution for time-series applications, as its training phase is fast and less computationally intensive, while also being immune to vanishing gradient problems that often plague deep feed-forward structures.

### AESNet-based node authentication and access decision model

The developed AESNet-based node authentication and access decision model is a security framework that is specially designed to safeguard VANET data transfers by enforcing strict access control for authorized nodes within blockchain architecture. In conventional Echo State Network (ESN), most of the internal dynamics are kept fixed after random initialization, and only the output weights are trained. When the term Adaptive Echo State Network (AESNet) is introduced, its adaptivity must therefore go beyond simple hyper-parameter tuning (e.g., spectral radius, leaking rate, or reservoir size). The justification for calling AESNet adaptive lies in its ability to self-modify its internal dynamical behavior in response to input data and environmental variations during operation. The reservoir computing framework in the AESNet model is more effective for capturing the temporal and dynamic behaviour of the vehicular communication data, unlike the traditional model. The real-time and complex varying patterns can be more effectively captured by the randomly connected reservoir, which acts as the dynamic memory. Based on the behavioural traits, the nodes are authenticated based on the time-varying patterns analyzed from this model. Furthermore, compared to the other models, the temporal and behavioural patterns of the nodes are analyzed using the AESNet, so the anomalies in the vehicular network are effectively determined using this model. Reservoir size is 800, spectral radius is 0.85, reservoir sparsity is 0.15 and here the initialization is done by the uniform, random and spectral scaling.

#### Node authentication

The developed AESNet is a deep learning architecture that was applied to identify authenticated nodes by analyzing their behaviour and identity traits. Key input attributes, such as the block header, previous block hash, merkle root, transaction details, and timestamp were fed into the AESNet model. This enabled accurate verification and classification of nodes within the network, enhancing security and reliability in data communication. These input attributes are collectively represented as $In_{T}^{BA}$. It is provided to the developed AESNet-based node authentication and access decision model. Firstly, the developed AESNet network ensures that every node of the blockchain whether it’s authenticated or not. The nature of the developed AESNet network plays a vital role in analyzing intricate features of the nodes for determining whether it is authenticated or not. This analysis is for facilitating the safe data transfer in the VANETs. In node authentication, it enhances the security of the VANET system by analyzing temporal patterns in node behaviour. Their reservoir structure captures dynamic input sequences of the provided attributes, allowing the system to detect anomalies or unauthorized access attempts based on subtle changes over time. This enables the developed AESNet to be particularly effective for authenticating blockchain nodes, where behaviour may vary but still follow identifiable patterns. With the results of the authentication, this network decides whether the data can be stored or not, to facilitate safe data transfer in VANETs.

#### Access decision making

If the nodes of the blockchain are authenticated, then the data can be stored in a particular node. Once a node is verified, the model makes access decisions using predefined policies, enhancing both security and efficiency. The nature of authentication analysis facilitated by the developed AESNet approach helps to prevent unauthorized access, insider threats, and data breaches by combining intelligent authentication with dynamic access control, making it especially useful in VANETs for secure communication among nodes without any security breaches. The performance of the authentication analysis for decision-making carried out by the developed AESNet approach is enhanced by the optimal parameter tuning like hidden node unit, system learning rate, and activation function, with proposed PUE-FLO heuristic strategy. The deployment of this heuristic scheme strengthens the node authentication and access decisions, improving accuracy, which is a critical aspect of data transfer in VANETs without any malicious interference. The necessity of fitness evaluation in developed AESNet node-access model is detailed in following section.

#### Objective function

An application of objective function in node authentication and access decision model is to improve the analysis of the authenticated nodes to transfer and store data without any malicious threats. The fitness evaluation for identifying the authenticated node relies on the maximization of the system accuracy in understanding the behaviours and identifying the nodes for data storage and transfers in VANETs. A fitness evaluation of implemented AESNet was represented on Eq. (5).

5 $$Ft_{NT}^{AE} = \mathop {\arg \,\max }\limits_{{\left\{ {AB_{ES}^{P} ,CD_{ES}^{Q} ,EF_{ES}^{R} } \right\}}} \left( {\frac{1}{{Wc_{Ac} }}} \right).$$

Here, the term $L_{Ac}$ represents the accuracy and the parameters such as hidden node unit $AB_{ES}^{P}$, system learning rate $CD_{ES}^{Q}$ and system activation function $EF_{ES}^{R}$ are evaluated for the fitness value assessment within the range of $\left[ {5,255} \right]$,$\left[ {0.01,0.99} \right]$ and $\left[ {5,255} \right]$. The accuracy $Wc_{Ac}$ is presented in Eq. (6).

6 $$Wc_{Ac} = \frac{{T_{Aa} + T_{Ab} }}{{T_{Aa} + T_{Ab} + F_{Ca} + F_{Cb} }}.$$

**Fig. 4:**
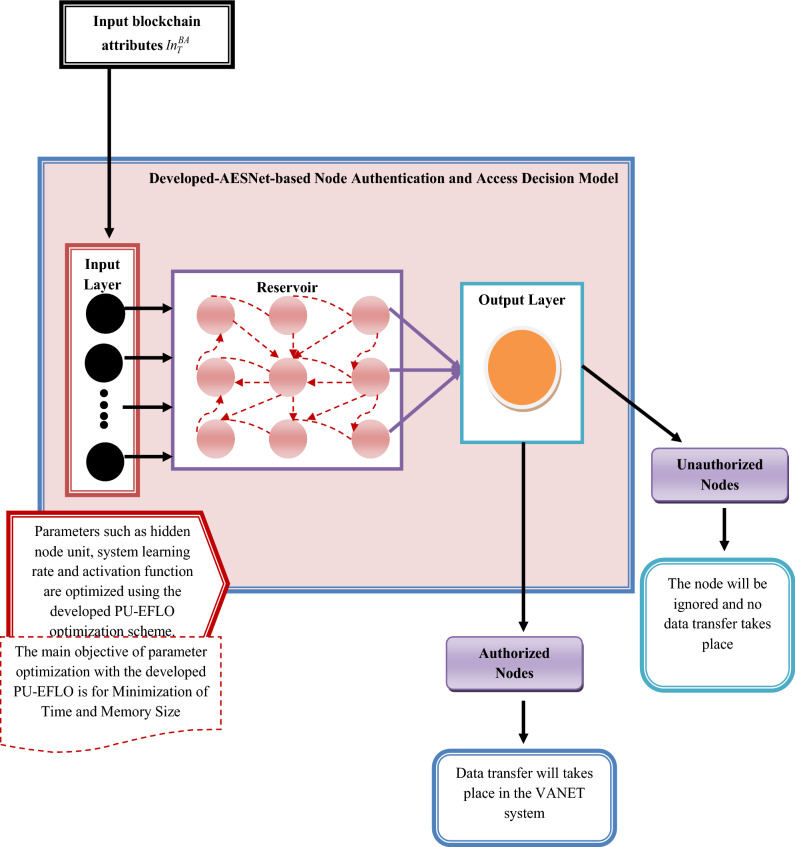
Schematic overview of AESNet-driven authentication system and access decision model.

Here, term $T_{Aa}$ is true positive, $T_{Ab}$ is true negative $F_{Ca}$ is false positive, and $F_{Cb}$ is false negative are used. An architectural illustration of developed AESNet-based node authentication and access decision methods was depicted at Fig. 4.

## Encryption and decryption for information-sharing using the developed optimal multikey generation-based cryptography technique

### Dual trapdoor homomorphic encryption

Dual Trapdoor Homomorphic Encryption^36^ is an advanced cryptographic technique that enhances data privacy by allowing computations on encrypted data while maintaining strong secrecy properties. It is built on the concept of trapdoor functions, which are easy to compute in one direction but hard to reverse without a secret key. In the case of a dual-trapdoor scheme, two distinct trapdoor functions are integrated to create a dual-layer encryption system. This dual structure enables secure and efficient processing of sensitive information, especially while managing large-scale data for analysis. Supporting homomorphic operations such as addition and multiplication that operate directly on ciphertext, it allows meaningful analysis without exposing the original data. The dual trapdoor approach improves both computational efficiency and resistance to attacks, making it a promising solution for privacy-preserving applications.

#### Encuryption

The consideration of an input $W \in M_{d}$ plain text data with the private and public keys such as *Pb* and $K = J_{Pb}$ respectively. A data point is randomly chosen in the range of $Rnd \in M_{d}$. Here, the ciphertext is mentioned as *W*. Then, the computation of ciphertext can be based on the following Eqs. (7), (8) and (9).

7 $$A = J^{K} \bmod \,d^{2}$$

8 $$B = \left( {1 + Wd} \right)J^{Rnd} \bmod \,d^{2}$$

9 $$\left[ W \right] = \left( {A,B} \right)$$

#### Decryption

The decryption procedure takes place by decrypting the ciphertext with the following Eq. (10).

10 $$B^{\prime} = B^{{\alpha {}_{1}}} \bmod d^{2} = \left( {1 + W{}^{\alpha 1}d} \right)K^{Rnd*\alpha 1} \bmod d^{2}$$

This is represented as the first iteration algorithm for decrypting the ciphertext in the first trapdoor, and the second trapdoor is mentioned in Eq. (11). Then, the decrypted plaintext can be written as in Eq. (12).

11 $$B^{\prime\prime} = B^{\alpha 2} \bmod d^{2} = \left( {1 + W{}^{\alpha 2}d} \right)K^{Rnd*\alpha 2} \bmod d^{2}$$

12 $$W = {\raise0.7ex\hbox{${\left( {B^{\prime}.B^{\prime\prime} - 1} \right)}$} \!\mathord{\left/ {\vphantom {{\left( {B^{\prime}.B^{\prime\prime} - 1} \right)} {d\,\bmod \,d^{2} }}}\right.\kern-0pt} \!\lower0.7ex\hbox{${d\,\bmod \,d^{2} }$}}$$

Here, the partial decryption algorithm for the dual trapdoor is defined as in Eqs. (13) and (14).

13 $$Part\,de^{\alpha 1} = \left( B \right).\left\{ {B,B^{\prime}} \right\}$$

14 $$Part\,de^{\alpha 2} = \left( B \right).\left\{ {B,B^{\prime\prime}} \right\}$$

#### Homomorphic property

If any ciphertext *W* is encrypted in the process of encryption with a similar public key, it can be represented as $\left[ {_{W} 1} \right].\left[ {_{W} 2} \right] = \left[ {_{W} 1 +_{W} 2} \right]$. The multiplicative homomorphic property is depicted in Eq. (15).

15 $$\left[ {_{W} 1 +_{W} 2} \right] = \left[ {_{W} 1} \right]^{{_{W} 2}}$$

This represents the combined encryption strategy of the dual-trapdoor homomorphic encryption.

### Encryption and decryption for information-sharing using the developed DTHE-OMK

In VANETs, secure and efficient data transmission is essential, as seamless information sharing plays a critical role in network performance and safety. The developed DTHE-OMK framework introduces a robust encryption and decryption mechanism, particularly tailored for VANET environments. The application of the DTHE-OMK cryptography tool leverages a dynamic threshold to ensure that only authorized VANET members can decrypt shared data, thereby enhancing confidentiality and resilience against unauthorized nodes in the blockchain-based systems.

#### Encryption

The developed DTHE-OMK strategy is initiated with the input provision of VANET attributes specifically the owner’s credentials private key and signature, the hash values, and owner’s public key, which is generally termed as $Jn_{N}^{VA}$. The DTHE technique represents advanced method for data encryption and decryption, utilizing multi-key generation framework to secure input information, which allows for a dynamic and secure keying structure adaptable to various data-sharing scenarios in blockchain networks.

#### Decryption

The primary trapdoor handles basic public-key encryption, secondary one grants ability to query and modify encrypted records without complete private-key decryption. This architecture is essential for privacy-preserving protocols, enabling VANET nodes to process and access blockchain-stored information without compromising data sensitivity. A homomorphic scheme enables operations on ciphertext that, when decrypted, yield the same results as if the operations had been performed on the plaintexts before encryption. This facilitates secure collaborative processing among vehicles and infrastructure in VANETs without exposing the provided input attribute data. The acquired outcomes of this process will be securely encrypted and decrypted data.

#### Optimal multi-key generation for data encryption and decryption

An optimal multi-key generation was not arbitrary, but is meticulously refined using the developed PUE-FLO optimization scheme. Here, the bit size of the multikey is 12 to 15 bits. This optimized state ensures that the encryption keys are not only secure but also optimal in terms of computational cost and latency. The developed DTHE-OMK relies on the dual trapdoor feature, which adds a layer of flexibility and control to the homomorphic encryption process. The optimal multi-key framework further enhances the system capability by allowing multiple entities to encrypt data with independently generated, interoperable public keys in the encryption mechanism. The decentralization of the trust factor minimizes single-point vulnerabilities and supports secure multi-party data aggregation. Meanwhile, the integration of the PUE-FLO optimization scheme ensures that the keys adapt to the VANET’s dynamic network conditions, optimizing private and public key attributes that exist in binary format. Each node can securely generate the key pair while maintaining compatibility with the broader encryption ecosystem.

**Fig. 5:**
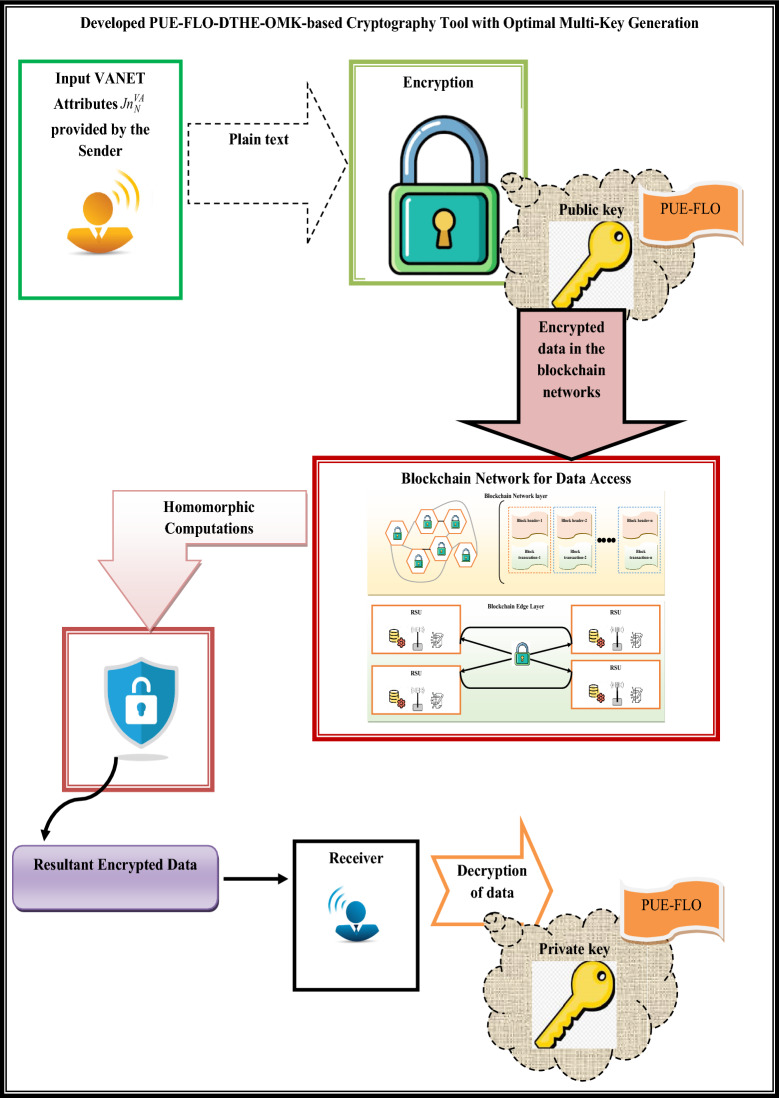
Pictorial presentation of encryption and decryption model for information-sharing using the developed DTHE-OMK cryptography tool.

Therefore, the developed PUE-FLO-DTHE-OMK-based encryption model with optimal multi-key generation offers highly resilient and scalable architecture for protected exchange of blockchain-stored information within VANET ecosystems. This method ultimately provides more secure and intelligent data transportation within the VANET systems without compromising data privacy and system performance. The pictorial presentation of the encryption and decryption model for information-sharing using the developed DTHE-OMK cryptography tool is provided in Fig. 5. Here, the input VANET attributes represent the data generated by the vehicles and that are passed to the encryption module for securely protecting the data. The optimized multikey cryptographic mechanism is used for encrypting the data; here, the optimal security performance is attained via the PUE-FLO-based tuning process. Within the blockchain system, the ciphertext is stored for secure and tamper-proof record keeping. Without decryption, the secure computation is directly performed on the encrypted data, and this process safeguards sensitive information during authentication and data-pooling operation.. The ciphertext is decrypted back to plain text by the private key that is developed using the PUE-FLO. Secure and reliable communication is performed by the authorized processing of the plain text data.

In our work, the DTHE-OMK cryptographic scheme builds upon the established dual trapdoor homomorphic encryption framework, which leverages the principles of homomorphic encryption with dual trapdoor structures to enable secure multi-party computation. The core equations and parameters from this prior scheme are reused to ensure robust privacy guarantees and functional correctness, specifically involving key generation, encryption, and decryption processes designed to support homomorphic operations on ciphertexts.

The primary novel contributions in our implementation focus on the integration of an optimized multi-key generation process and adaptive parameter tuning. We apply the PUE-FLO optimization algorithm to fine-tune key attributes, such as public and private key parameters, in both binary and real formats, across dynamic network conditions. This adaptive multi-key generation, guided by our optimization framework, ensures that each node independently generates interoperable key pairs that adapt to the VANET environment’s topology and security requirements. Consequently, our enhancements facilitate scalable, secure, and efficient multi-party encryption, enabling seamless collaborative data processing while maintaining the cryptographic strength provided by the foundational DTHE-OMK scheme.

### Objective function

The performance optimization is paramount in the developed PUE-FLO-DTHE-OMK-based information sharing system. An objective function is specifically modeled to curtail latency and buffer requirements during cryptographic cycles. These constraints are vital in bandwidth-limited VANETs, where computational agility is prerequisite for seamless network communication. The objective function of the encryption and decryption for information-sharing using the developed DTHE-OMK is depicted in Eq. (16).

16 $$Ft_{DT}^{ED} = \mathop {\arg \,\min }\limits_{{\left\{ {\Pr i_{DT}^{AB} ,Pub_{DT}^{CD} } \right\}}} \left( {L_{t} + L_{MS} } \right).$$

Here, the terms $\Pr i_{DT}^{AB}$ and $Pub_{DT}^{CD}$ are the private and public keys that are present in the binary format [0, 1].The time consumption is referred to as $L_{t}$ and $L_{MS}$ is the memory size of the system. The Eq. (17) and (18) represent the time consumption $L_{t}$ and $L_{MS}$ memory size, respectively.

17 $$L_{t} = Encr_{T} + Decr_{T} + Ovr_{T} ,$$

18 $$L_{MS} = H_{Tot} \times H_{size} .$$

Here, the term $Encr_{T}$ is the encryption time, $Decr_{T}$ decryption time,$Ovr_{T}$ overhead time and $H_{Tot}$ is the total count of data units with a size per unit $H_{size}$.

In the proposed optimization-driven AESNet authentication framework, parameter tuning is structured as multi-objective optimization problem, requiring multiple performance criteria must be simultaneously optimized. These objectives are inherently conflicting in nature: improving authentication accuracy may increase computational complexity, thereby increasing latency, while minimizing latency may compromise verification robustness. Hence, a unified scalar objective function is required to enable efficient optimization using metaheuristic algorithms. When these disparate measurements are directly combined, scale bias is introduced, wherein metrics with greater numerical magnitude (such as latency) control the optimization process regardless of their true significance. Min–max normalization is used to address these issues.

## Results and discussion

### Experimental evalauation

A secure blockchain-based authentication and data transfer system in VANETs was evaluated using Python-based simulations. All performance gains reported in the experimental and ablation analyses correspond to absolute percentage point improvements, not relative percentage increases. The proposed PUE-FLO-AESNet framework was assessed using optimization algorithms, including Carpet Weaver Optimization (CWO)^37^, Three on Three Optimizer (TOT)^38^, Wombat Optimization Algorithm (WOA)^39^, and FLO^34^. Its performance was compared against deep learning models like DNN^40^, LSTM^41^, GRU^22^, and ESNet^35^ to validate authentication accuracy. For encryption, classical and modern techniques such as RSA^42^, DES^43^, ECC^44^, and DTHE-OMK^36^ were used. The results confirmed the system’s effectiveness in enhancing secure data transmission across VANET environments. A 3 km × 3 km urban road network topology is covered by 12 Road Side Units (RSUs) and 150 vehicle nodes in VANET simulation scenario. A Manhattan Grid Mobility mimic simulates real-world urban traffic dynamics, such as crossings, bidirectional lanes, acceleration-deceleration patterns, and traffic signal disruptions, in order to mimic vehicle mobility. To reflect the varied traffic situations commonly found in cities, the vehicle speed ranges from 20 km/h to 80 km/h. According to IEEE 802.11p communication standards, vehicular nodes function with a transmission range of 150 m, whereas each RSU is presumed to have fixed infrastructure support with a communication range of 300 m. All optimization algorithms employed in the study are configured with an identical computational budget to facilitate a fair comparison. Specifically, each algorithm operates with population size 50 agents, maximum 100 iterations, and total fitness evaluation limit set to 5,000 evaluations. Similarly, the neural models, including DNN, LSTM, GRU, and ESNet are trained under standardized parameters: learning rate 0.001, total 100 epochs, and batch size 64. This uniform configuration ensures that the comparative performance assessments of the optimization techniques and neural models are conducted under consistent resource constraints, thereby providing an equitable basis for analyzing their respective efficiencies and effectiveness in the system.

A controlled adversarial model is added to the simulation environment to assess the robustness of authentication:

Vehicles that adhere to encrypted data exchange protocols and protocol-compliant authentication are considered as legitimate nodes. Vehicles that purposefully break authentication procedures are known as malicious nodes. They do this by:

- Broadcasting impersonation attacks using fake identity credentials,
- Introducing modified safety messages (attack via data injection),
- Attempting to access RSU communication channels without authorization (Sybil Behaviour).

Here, 15% of all vehicles are malicious nodes, which are inserted into the network at random intervals throughout the simulation runtime. These nodes generate falsified transaction signatures or encrypted packets to disrupt secure message dissemination.

### Performance metrics

The evaluation metrics related to secure authentication and information sharing are presented in the following section:

(a) Precision $Wp_{pre}$ in Eq. (19)

19 $$Wp_{pre} = \frac{{T_{Aa} }}{{T_{Aa} + F_{Ca} }}.$$

(b) F1-Score $W_{F1}^{scr}$ in Eq. (20).

20 $$W_{F1}^{scr} = \frac{{2 \times \left( {T_{Aa} } \right)}}{{2 \times \left( {T_{Aa} + F_{Ca} + F_{Cb} } \right)}}.$$

(c) False Positive Rate (FPR)$WR_{fp}$ in Eq. (21).

21 $$WR_{fp} = \frac{{F_{Cb} }}{{F_{Cb} + T_{Ab} }}.$$

(l) Total computation time *TcT* is represented in Eq. (22)

22 $$TcT = \sum\nolimits_{m} {\frac{{LT_{j} }}{{CPU_{rj} }}} .$$

Here, the term $LT_{j}$ is the total length of the task instructions and $CPU_{rj}$ is the rate of the CPU performance.

(m) Encryption time *EnT* is presented in Eq. (23)

23 $$EnT = \frac{{AD_{size} }}{{H_{speed} }}.$$

Here, the term $H_{speed}$ is the speed and $AD_{size}$ is the total data size.

(n) Decryption time *DcT* is represented in Eq. (24)

24 $$DcT = A_{os} \times A_{T} \times S_{ovf} .$$

Here, the term $A_{os}$ is the number of operations,$A_{T}$ is the time per operation and $S_{ovf}$ is the system overhead.

(o) Memory size $M_{sz}$ is in Eq. (25).

25 $$M_{sz} = \frac{{A_{n} }}{DB}$$

Here, the term *DB* is the bytes per unit and $A_{n}$ is the total number of data.

(p) Key sensitivity analysis $K_{SA}$ is provided in Eq. (26)

26 $$K_{SA} = A_{v} S = \frac{{T_{NCT} }}{{T_{c} }} \times 100\% .$$

Here, the term $T_{NCT}$ indicates number of modified ciphertext bits and $T_{c}$ total bit volume. The avalanche effect ratio expressed as a percentage is denoted by $A_{v} S$.

### Simulation parameters

Table 3 provides the simulation parameters used in the developed model.

**Table 3:** Simulation parameters.

Cryptography parameters	
Vehicle ID	VID001–VID1000
Vehicle type	Car
Position (GPS)	(39.5421
Speed	10–120 km/h
Direction	0°–360°
Neighbor count	3–25
Communication range	250–500 m
Transmission power	10–20 dBm
Mobility model	Random Waypoint
Node density	20–100 nodes/km^2^
Authentication parameters	
Owner’s private key	2048-bit RSA/256-bit ECC
Owner’s public key	256-bit ECC Public Key
Owner’s signature	SHA-256/ECDSA Signature
Hash	256-bit Secure Hash (SHA-256)
Transaction contents	Encrypted Message/Data Block
Timestamp	UNIX/UTC Time
Previous hash	256-bit Hash Value
Next owner’s public key	256-bit ECC Public Key
Authentication token	128-bit Session Token
Access decision (AESNet output)	0 (Reject)/1 (Accept)
Trust score	0.0–1.0
Certificate	X.509 Digital Certificate
Digital signature	ECDSA/RSA Signature
Nonce	32-bit Random Integer
Encryption key	128/256-bit Symmetric Key (DTHE-OMK)
Decryption key	Corresponding Private Key
Message digest	256-bit
Merkle root	256-bit Hash
Block ID	B0001–B0100
Verification key	ECC/RSA Public Key
Authentication token	128-bit Session Token
Access decision (AESNet output)	0 (Reject)/1 (Accept)

### Cost function analysis of proposed approach

**Fig. 6:**
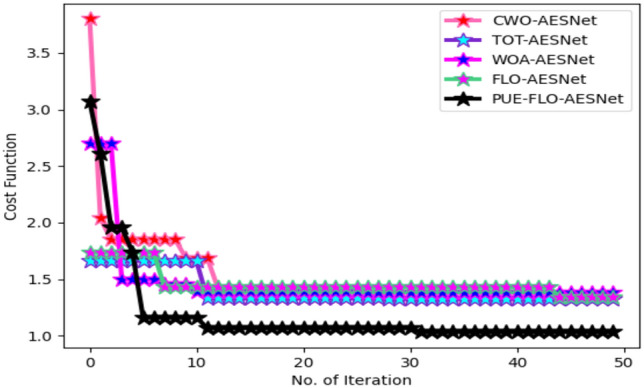
Cost function of implemented system.

The stability and dependability of proposed architecture were verified through cost function analysis illustrated in Fig. 6. To evaluate convergence behavior, number of training iterations is plotted along horizontal axis, while cost values are represented on vertical axis. A performance in PUE-FLO-AESNet was tested using different optimization algorithms, including CWO-AESNet, TOT-AESNet, WOA-AESNet, and FLO-AESNet. This comparison provided insights into the model’s robustness and adaptability across various tuning strategies. By evaluating the convergence value, it models efficiency and can benchmark its performance against existing methodologies in the range of 75.000%, 66.667%, 61.538% and 37.500% of CWO-AESNet, TOT-AESNet, WOA-AESNet, and FLO-AESNet, respectively. Therefore, the lower convergence value of the developed PUE-FLO-AESNet model is indicative of enhanced performance, especially in tasks involving pattern formation and discovery, delivering results with high accuracy outcomes.

### Varying epoch-based node authentication validation

**Fig. 7:**
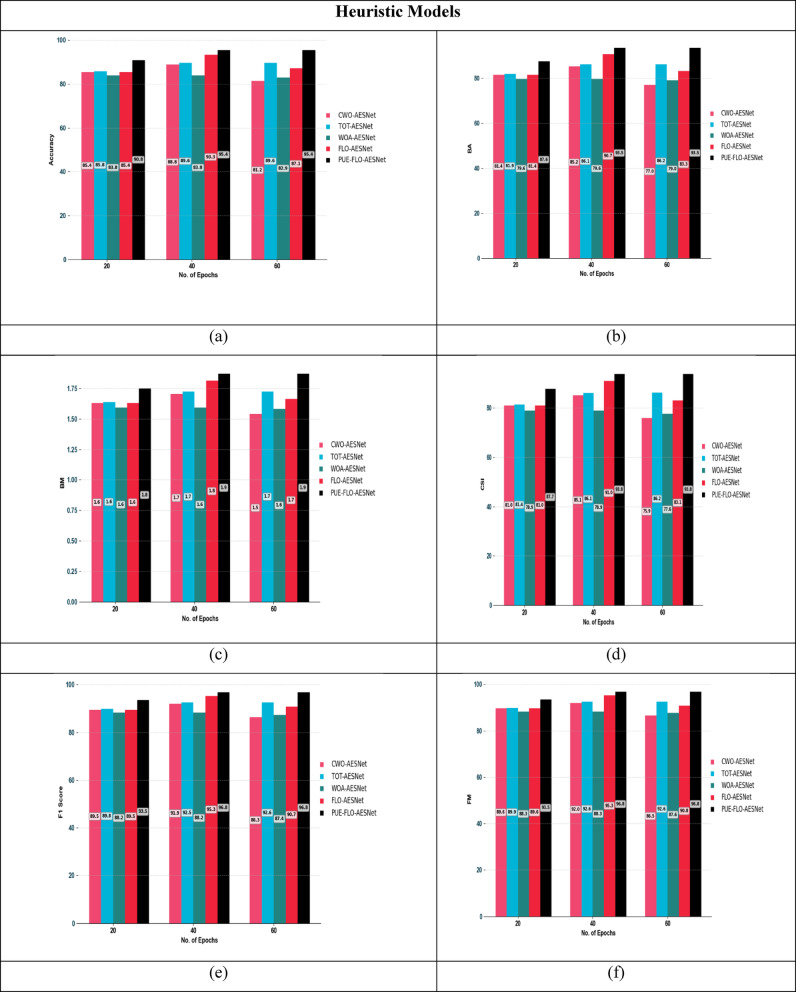

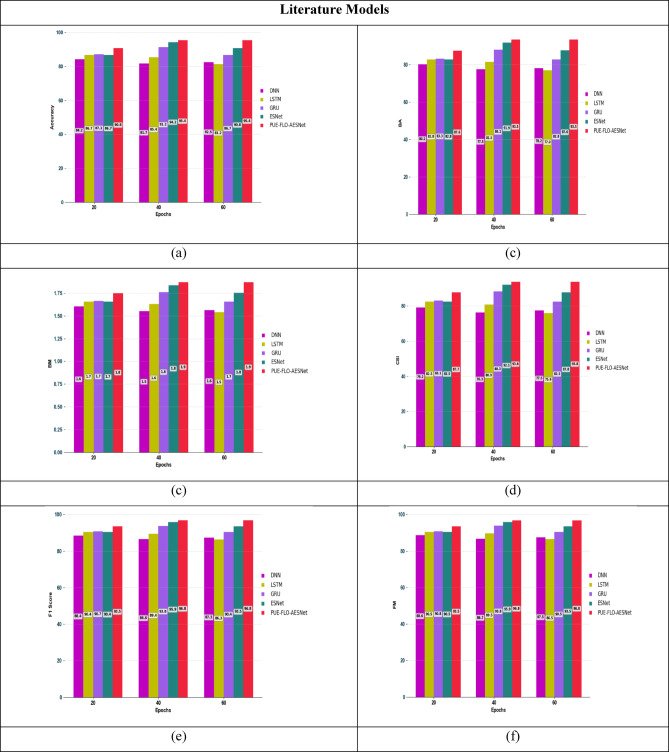
Node authentication analysis of implemented system on varying epochs for various optimization strategies and techniques in terms of “(**a**) Accuracy, (**b**) BA, (**c**) BM, (**d**) CSI, (**e**) F1 Score, (**f**) FM”.

Figure 7 illustrates node authentication analysis on varying epochs for various heuristics and literature models. In this representation, horizontal axis denotes epoch count, while vertical axis displays performance metrics, with values expressed as percentages. This analysis identifies risk-associated areas and highlights improvements in user experience in the VANETs through enhanced security mechanisms. The developed PUE-FLO-AESNet is evaluated with the optimization algorithms such as CWO-AESNet, TOT-AESNet, WOA-AESNet, and FLO-AESNet. The analysis of the accuracy present in Fig. 7a suggest that PUE-FLO-AESNet was highly reliable at authentication and decision-making task, with the values of 6.323%, 5.828%, 8.353% and 6.323% for the optimization algorithms such as CWO-AESNet, TOT-AESNet, WOA-AESNet, and FLO-AESNet, and 7.838%, 4.729%, 4.248%, and 4.729% for DNN, LSTM, GRU, and ESNet, respectively. The balanced accuracy of the model is 93.5%, the BM of the model is 1.9, the CSI of the model is 93.8, the F1 score of the model is 96.8, FM of the model is 96.8.These results confirm that the PUE-FLO-AESNet model is a significant secured data transfer in VANET with accurate node authentication outcomes within blockchain-based systems. Thus, the model ensures accurate node verification with improved behavioural patterns analysis.

### ROC analysis of proposed framework

**Fig. 8:**
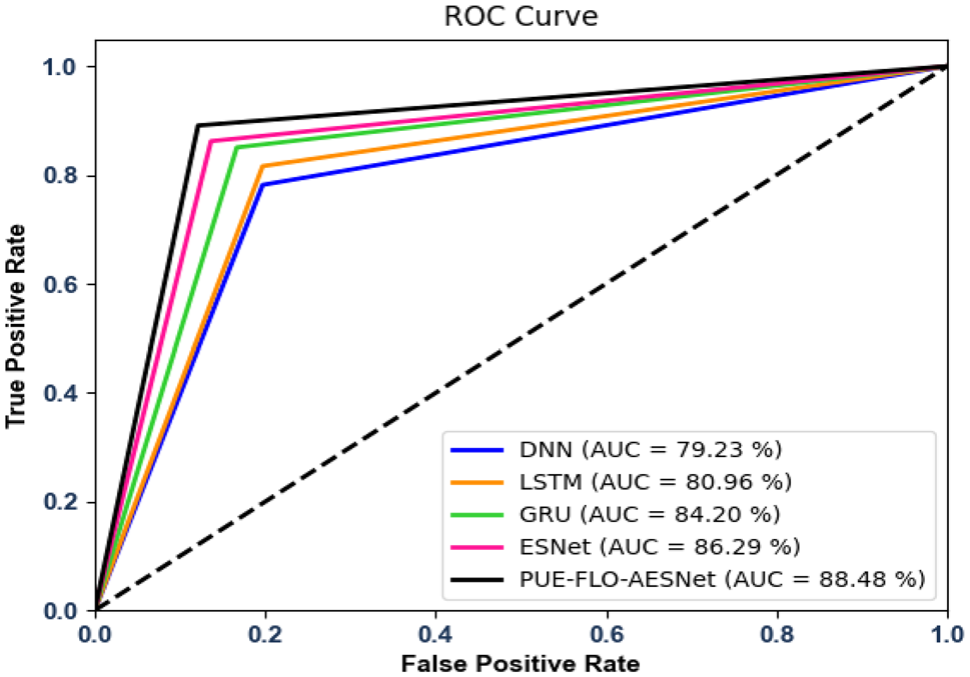
ROC Assessment of ROC.

ROC characteristics for proposed blockchain-integrated deep learning framework are illustrated in Fig. 8. This evaluation maps FPR along horizontal axis against TPR on vertical axis. To benchmark system’s authentication efficacy, the model was compared against established architectures, including DNN, LSTM, GRU, and ESNet. Experimental results indicate that developed model outperformed these counterparts by margins 11.675%, 9.289%, 5.033%, and 2.538%, respectively. Such improvements underscore framework’s superior capability in access decision-making while maintaining enhanced privacy standards. Thus, the developed PUE-FLO-AESNet model supports real-time decision-making for routing strategies, especially in dynamic environments with frequent topology changes and high mobility.

### Node authentication of implemented approach using activation function

Tables 4 and 5 illustrate how different activation functions impact authentication within various metaheuristic and deep learning models. By analyzing ReLU, TanH, and softmax among others for models like CWO-AESNet and ESNet, the study provides clear comparative analysis. These results highlight robustness of framework in managing node authorization and ensuring secure communication within network. The analysis of the FPR for Tanh depicts that PUE-FLO-AESNet demonstrate highly reliable result at authentication and decision-making task, with the values of 42.212% of CWO-AESNet, 61.861% of TOT-AESNet, 8.162% of WOA-AESNet, 48.534% of FLO-AESNet and 47.419% of DNN, 61.350% of LSTM, and 55.801% of GRU, respectively. These results confirm that the PUE-FLO-AESNet model is a significant secured data transfer in VANET with accurate node authentication outcomes within blockchain-based systems. Thus, this evaluation serves as a crucial foundation to assess integrity and throughput potential of model within authentic, large-scale environments.

**Table 4:** Node authentication analysis of the implemented approach based on different activation functions for various metaheuristic algorithms.

Activation function	CWO-AESNet^37^	TOT-AESNet^38^	WOA-AESNet^39^	FLO-AESNet^34^	PUE-FLO-AESNet
Accuracy					
Linear	85.6	89.0	86.3	89.4	93.4
Tanh	87.6	81.1	92.2	86.2	92.8
ReLu	77.9	89.4	90.0	88.3	92.1
Softmax	82.5	79.9	86.7	86.5	91.8
Sigmoid	84.2	89.8	81.8	91.8	94.0
Specificity					
Linear	85.5	89.2	86.5	89.3	93.3
Tanh	87.5	81.1	92.1	86.0	92.8
ReLu	77.9	89.4	89.9	88.3	92.3
Softmax	82.5	79.8	86.3	86.3	91.8
Sigmoid	83.9	89.6	81.8	91.8	93.9
FPR					
Linear	14.5	10.8	13.5	10.7	6.7
Tanh	12.5	18.9	7.9	14.0	7.2
ReLu	22.1	10.6	10.1	11.7	7.7
Softmax	17.5	20.2	13.7	13.7	8.2
Sigmoid	16.1	10.4	18.2	8.2	6.1
NPV					
Linear	85.7	88.6	86.1	89.6	93.4
Tanh	87.7	81.1	92.1	86.5	92.8
ReLu	77.9	89.3	90.1	88.3	91.8
Softmax	82.4	80.0	87.2	86.7	91.7
Sigmoid	84.6	89.9	81.6	91.7	94.1
MCC					
Linear	0.7	0.8	0.7	0.8	0.9
Tanh	0.8	0.6	0.8	0.7	0.9
ReLu	0.6	0.8	0.8	0.8	0.8
Softmax	0.7	0.6	0.7	0.7	0.8
Sigmoid	0.7	0.8	0.6	0.8	0.9
BM					
Linear	1.7	1.8	1.7	1.8	1.9
Tanh	1.8	1.6	1.8	1.7	1.9
ReLu	1.6	1.8	1.8	1.8	1.8
Softmax	1.7	1.6	1.7	1.7	1.8
Sigmoid	1.7	1.8	1.6	1.8	1.9

**Table 5:** Node authentication analysis of the implemented approach-based on different activation functions for various literature models.

Activation function	DNN^40^	LSTM^41^	GRU^22^	ESNet^35^	PUE-FLO-AESNet
Accuracy					
Linear	85.6	88.6	81.0	89.3	93.4
Tanh	86.5	81.5	83.4	92.8	92.8
ReLu	87.4	84.8	89.8	90.1	92.1
Softmax	78.5	80.6	84.3	87.0	91.8
Sigmoid	82.3	86.2	91.8	92.4	94.0
Specificity					
Linear	85.8	88.6	80.6	89.3	93.3
Tanh	86.3	81.3	83.7	92.8	92.8
ReLu	87.4	84.7	89.6	90.1	92.3
Softmax	78.6	80.4	84.5	87.1	91.8
Sigmoid	82.6	86.1	91.7	92.3	93.9
FPR					
Linear	14.2	11.4	19.4	10.7	6.7
Tanh	13.7	18.7	16.3	7.2	7.2
ReLu	12.6	15.3	10.4	9.9	7.7
Softmax	21.4	19.6	15.5	12.9	8.2
Sigmoid	17.4	13.9	8.3	7.7	6.1
NPV					
Linear	85.3	88.6	81.4	89.3	93.4
Tanh	86.7	81.7	83.0	92.8	92.8
ReLu	87.5	84.9	89.9	90.1	91.8
Softmax	78.2	80.8	84.0	86.9	91.7
Sigmoid	81.9	86.4	92.0	92.5	94.1
MCC					
Linear	0.7	0.8	0.6	0.8	0.9
Tanh	0.7	0.6	0.7	0.9	0.9
ReLu	0.7	0.7	0.8	0.8	0.8
Softmax	0.6	0.6	0.7	0.7	0.8
Sigmoid	0.6	0.7	0.8	0.8	0.9
BM					
Linear	1.7	1.8	1.6	1.8	1.9
Tanh	1.7	1.6	1.7	1.9	1.9
ReLu	1.7	1.7	1.8	1.8	1.8
Softmax	1.6	1.6	1.7	1.7	1.8
Sigmoid	1.6	1.7	1.8	1.8	1.9

### Security analysis of proposed approch

**Fig. 9:**
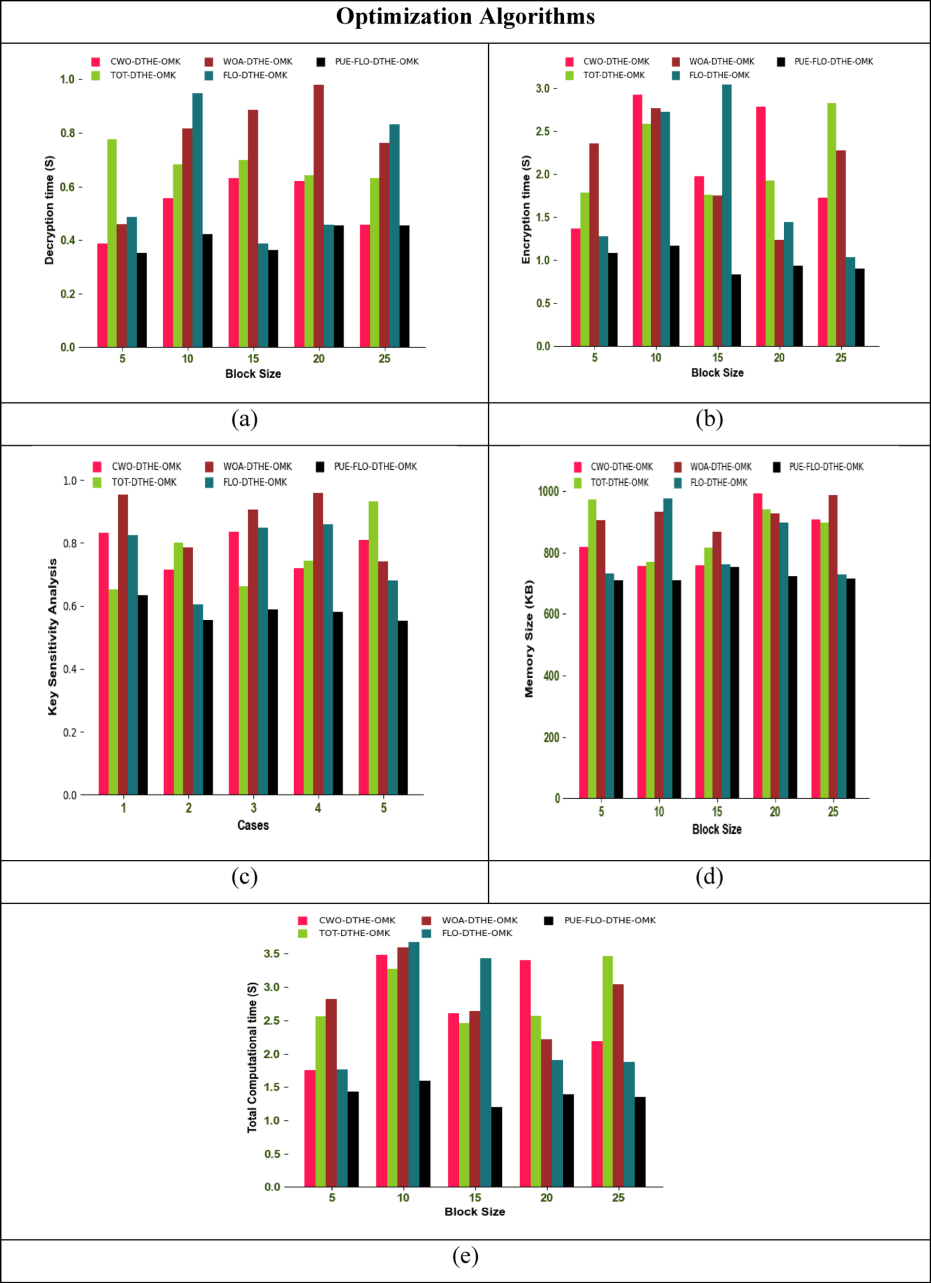

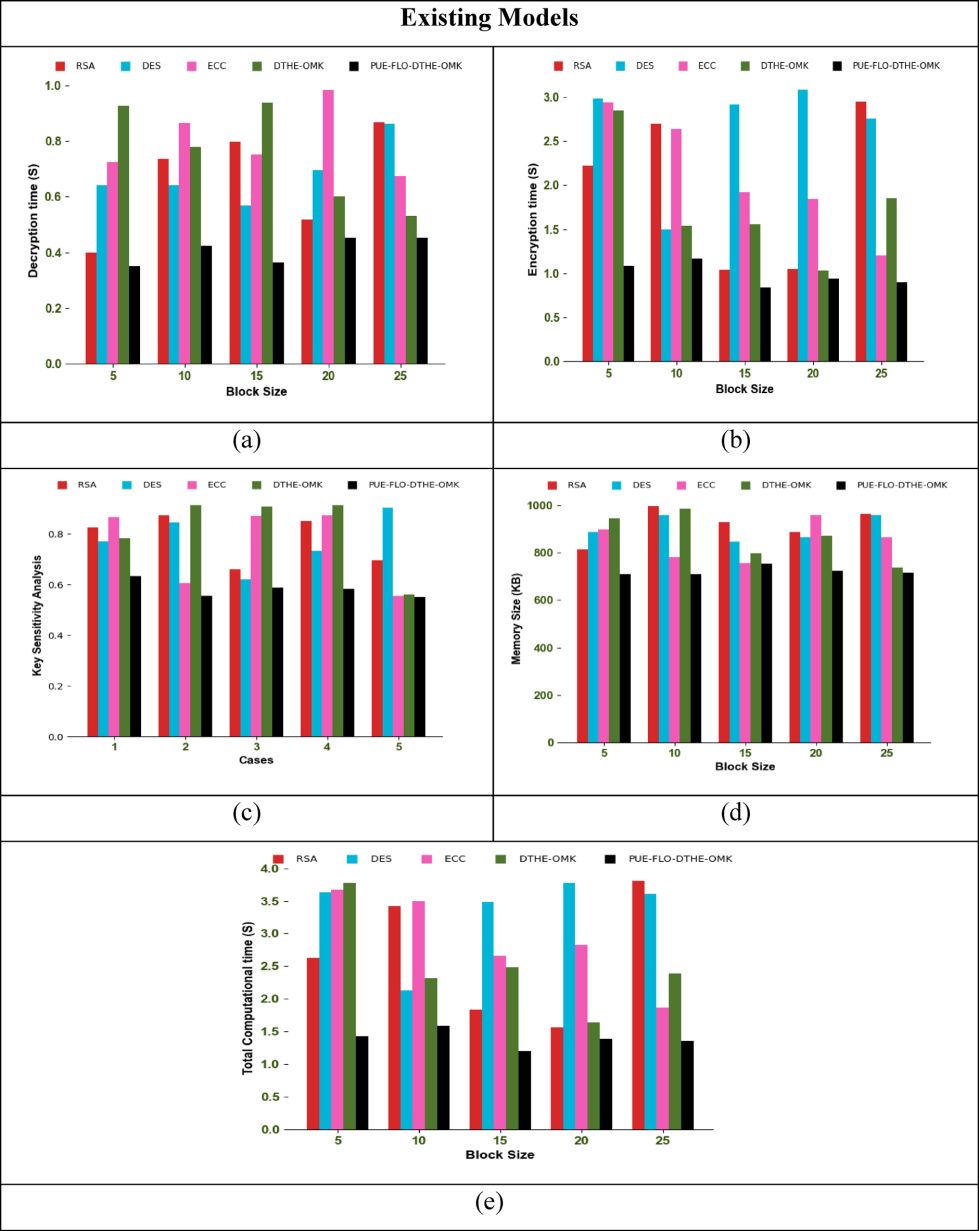
Encryption assessment of Implemented Approach for existing optimizers and Existing techniques based on “(**a**) Decryption Time, (**b**) Encryption Time, (**c**) Key Sensitivity Analysis, (**d**) Memory Size, and (**e**) Total Computation Time”.

An assessment of encryption efficiency is illustrated in Fig. 9. The X-axis represents data block size, while Y-axis indicates computational costs, such as encryption and decryption latency, memory utilization, and overall execution time. These measurements are quantified in seconds and memory units. This helps to ensure robustness of data protection and secure communication assured by the implemented approach with integration of advanced PUE-FLO-DTHE-OMK encryption mechanisms tailored to the operational demands of the target VANET application. This mechanism is effective with the values of 30.000% of RSA, 26.316% of DES, 22.222% of ECC, and 6.667% of DTHE-OMK and shows that the developed PUE-FLO-DTHE-OMK approach utilizes a low memory size compared to other models. The suggested framework achieves rapid encryption speeds, maintaining low computational footprint during data protection tasks. The total computation time, memory size and key sensitivity are also low for the designed model. Therefore, this depicts that the ability to resist cryptographic attacks by maintaining low computational overhead can preserve data integrity across diverse communication scenarios with improved robustness.

### Statistical assessment of implemented approach

The statistical evaluation of blockchain-driven deep learning VANET systems for authenticated data transfers is depicted in Table 6. This assessment is a very critical process used to assess how securely, accurately, and rapidly system handles encrypted information exchange. A worst factor analysis depicts that the model is highly accurate in detecting the malicious node in data transfer with the values of 19.184%of CWO-AESNet, 85.000% of TOT-AESNet, 13.909% of WOA-AESNet, and 77.003%of FLO-AESNet. These results proved that the scalability and blockchain overhead can be enhanced to guarantee performance under growing data volumes and vehicular density. As a result, proposed PUE-FLO-AESNet architecture validate feasibility of large-scale deployment without compromising network’s resilience or confidentiality protocols.

**Table 6:** Statistical assessment of implemented approach.

TERMS	CWO-AESNet^37^	TOT-AESNet^38^	WOA-AESNet^39^	FLO-AESNet^34^	PUE-FLO-AESNet
BEST	1.3	1.3	1.4	1.3	1.0
WORST	3.8	1.7	2.7	1.7	3.1
MEAN	1.5	1.4	1.5	1.5	1.2
MEDIAN	1.3	1.3	1.4	1.4	1.1
STD	0.4	0.1	0.3	0.1	0.4

### Analytical comparison of suggested approach

An analytical comparison in PUE-FLO-AESNet is given in Table 7. Here, the comparison is done by considering the accuracy, specificity and false positive metrics with the existing models. As per the results mentioned in Table 7, the suggested PUE-FLO-AESNet model attained the accuracy value of 94%, specificity of 92.91% and false positive rate of 7.09% at the sigmoid activation function. This analytical outcome of the suggested PUE-FLO-AESNet model is more effective to confirm how effectively PUE-FLO-AESNet manages secure identity authentication.

**Table 7:** Analytical comparison of the developed model.

Activation function	DNN^40^	LSTM^41^	GRU^22^	ESNet^35^	PUE-FLO-AESNet
Accuracy					
Linear	87.9	82.2	88.6	93.4	
Tanh	82.9	85.0	91.5	92.8	
ReLU	85.4	90.5	91.2	92.1	
Softmax	81.9	85.3	88.2	91.8	
Sigmoid	87.5	92.5	93.1	94.0	
Specificity					
Linear	84.9	88.1	81.2	88.8	
Tanh	85.5	82.6	84.1	91.4	
ReLU	87.9	85.2	90.2	91.0	
Softmax	79.9	81.5	85.8	88.6	
Sigmoid	83.2	87.0	92.2	92.9	
False positive rate					
Linear	15.1	11.9	18.8	11.2	
Tanh	14.5	17.4	15.9	8.6	
ReLU	12.1	14.8	9.8	9.0	
Softmax	20.1	18.6	14.2	11.4	
Sigmoid	16.8	13.0	7.8	7.1	

### Ablation experiment on individual components

The ablation analysis is given in Table 8 to prove the contribution of each component in the suggested authentication process. The standard ESNet model achieved initial authentication of 91.24% accuracy. However, integration of blockchain technology and PUE-FLO parameter optimization significantly boosted performance, reaching a peak accuracy of 99.14%. These findings confirm that AESNet and DTHE-OMK encryption scheme facilitates highly secure communication within vehicular networks. Under the dynamic topology conditions, the PUE-FLO is proposed for parameter tuning in the authentication framework. The privacy preservation, trust management and node verification are attained through the unified authentication and encryption framework. Further, the data tampering attacks in the decentralized environment are effectively solved through the enhanced optimized secure communication model proposed in this work. Unlike the traditional framework, the suggested model can effectively perform the trust management in the decentralized framework. In addition, the recommended model also addresses the insufficient privacy preservation at the time of data exchange.

**Table 8:** Ablation experiment.

Model configuration	Accuracy (%)
Baseline ESNet	91.2
AESNet	93.7
AESNet + DTHE-OMK	95.1
AESNet + Blockchain	94.8
AESNet + PUE-FLO	96.5
AESNet + DTHE-OMK + Blockchain	97.6
AESNet + DTHE-OMK + PUE-FLO	98.1
AESNet + Blockchain + PUE-FLO	97.7
Proposed model (full integration)	99.1

### Statistical results of the meta-heuristic algorithms and models/results with confidence interval

Table 9 displays accuracy analysis of PUE-FLO-AESNet model with confidence interval and standard deviation. The results showed the recommended model attained the accuracy of 93.99% with minimum standard deviation of 0.48, which validates efficacy of proposed PUE-FLO-AESNet framework within identity verification phase. Likewise, the statistical report of the metaheuristic algorithm is given in the Table 9 that shows the accuracy of the suggested AESNet + PUE-FLO model is 99.1 with the standard deviation of 0.42 that shows the effective performance of the metaheuristic algorithm in optimization process.

**Table 9:** Results with standard deviation/confidence interval.

Model	Mean (%)	Std. dev	95% Confidence interval
Accuracy			
DNN	84.9	1.4	[84.38, 85.44]
LSTM	87.3	1.2	[86.82, 87.70]
GRU	90.1	1.1	[89.73, 90.55]
ESNet	92.1	1.0	[91.72, 92.44]
PUE-FLO-AESNet	94.0	0.5	[93.81, 94.17]

Meta-heuristic algorithm			
Model variant	Accuracy (%)	Std. dev. (σ)	95% Confidence interval
AESNet + WOA	96.1	± 0.84	[95.38–96.86]
AESNet + FLO	96.9	± 0.71	[96.31–97.57]
AESNet + TOT	97.2	± 0.66	[96.64–97.78]
AESNet + CWO	97.4	± 0.59	[96.84–97.86]
AESNet + PUE-FLO	99.1	± 0.42	[98.77–99.51]

### Security level and key size in encryption scheme

Table 10 details key size and security level of implemented method against traditional approaches. As per the results from Table 10, the recommended model attained the security level of 128 bits with the key size of 256 bits. This outcome revealed that the proposed model requires small key size for the encryption process, but it yields substantially greater security for transmitted data.

**Table 10:** Key size and security level of encryption models.

Encryption scheme	Key size	Security level
RSA-based HE	2048-bit	112-bit
ECC-based scheme	256-bit	128-bit
Paillier encryption	3072-bit	128-bit
DTHE-OMK (proposed)	256-bit	128-bit

### Mapping of design parameters to real-time VANET constraints and safety–critical latency requirements

Table 11 shows the design parameters to real-time VANET constraints and safety–critical latency requirements. This table provides the details regarding the components of the VANET and its safety constraints, and verifies whether it meets the requirements or not. As per the analysis, most of the metrics meet the requirements and also provides satisfied level of safety in the VANET communication process.

**Table 11:** Design parameters to real-time VANET constraints and safety–critical latency requirements.

Component	Parameter choice	Measured/estimated time	VANET safety constraints	Meets requirement
Vehicle-to-RSU authentication	AESNet inference (reservoir size = 300)	6.8 ms	≤ 100 ms (authentication delay)	Yes
Parameter optimization	PUE-FLO (offline tuning)	Offline (not real-time)	No real-time constraint	Yes
Blockchain verification	PBFT consensus (10–20 RSUs)	18–25 ms	≤ 50 ms (trust validation)	Yes
Block generation	Block interval = 1–2 s	Background process	Non-safety–critical	Yes
Encryption (proposed)	DTHE-OMK (128-bit equivalent)	9.4 ms (encrypt + decrypt)	≤ 100 ms (secure message)	Yes
Encryption (baseline RSA)	RSA-2048	21.6 ms	≤ 100 ms	Yes
Encryption (baseline FHE)	BFV/BGV	65–85 ms	≤ 100 ms	Marginal
End-to-end authentication delay	Auth + encryption + verification	38–45 ms	≤ 100 ms (safety apps)	Yes
Message dissemination	IEEE 802.11p	2–5 ms	≤ 20 ms	Yes
Emergency message handling	Authentication bypass cache	< 10 ms	≤ 50 ms (collision warning)	Yes

### Formal security proof

The formal proof of security for implemented model is summarized in Table 12. This analysis is done by taking authentication accuracy, attack detection rate, false positive rate, convergence time, and security stability index. The presented PUE-FLO–AESNet model reaches the authentication accuracy of 98.43%, attack detection rate of 97.96% and false positive rate of 1.86 that shows the effective performance of the model in the authentication process.

**Table 12:** Formal security proof of the developed model.

Models	Authentication accuracy	Attack detection rate	False positive rate	Convergence time	Security stability index
CWO-AESNet	92.14	91.78	6.42	21.6	0.89
TOT-AESNet	92.63	92.1	6.01	20.4	0.91
WOA-AESNet	90.38	89.92	7.33	23.8	0.87
FLO-AESNet	92.14	91.85	6.35	19.7	0.92
PUE-FLO–AESNet	98.43	97.96	1.86	14.3	0.97

### Comparison of activation functions with existing approaches

A comparison against activation functions with existing and proposed methods is discussed in Table 13. The proposed system utilizes Consortium (Permissioned) blockchain with Practical Byzantine Fault Tolerance (PBFT) consensus mechanism for managing authentication across vehicles, RSUs, and edge nodes. The proposed approach integrates an AESNet, a deep learning framework for authentication and DTHE-OMK, a homomorphic encryption for secure data processing. When compared against existing models, like DNN, LSTM, GRU, and standard ESNet, the proposed PUE-FLO-AESNet demonstrates superior performance across various activation functions. Specifically, using the sigmoid function, developed framework recorded highest accuracy of 94% and specificity of 92.9%, while significantly reducing the FPR to 7.1%. These findings confirm that the proposed blockchain-enabled architecture outperforms current methods by providing a more reliable and precise security framework for VANET environments.

**Table 13:** Comparison of activation functions with existing approaches.

Activation-function	DNN^40^	LSTM^41^	GRU^22^	ESNet^35^	Proposed PUE-FLO-AESNet
Accuracy					
Linear	85.92	81.04	85.36	84.24	93.4
Tanh	83.84	83.44	92.88	87.28	92.8
ReLU	83.44	88.72	87.84	89.84	92.1
Softmax	77.52	82.72	82.32	85.04	91.8
Sigmoid	81.68	91.28	91.36	92.24	94
Specificity					
Linear	85.76	80.48	85.14	83.90	88.8
Tanh	83.44	83.20	92.87	86.84	91.4
ReLU	83.20	88.65	87.70	89.66	91
Softmax	77.22	82.52	81.96	85.15	88.6
Sigmoid	81.32	91.11	91.13	92.08	92.9
FPR					
Linear	14.24	19.52	14.86	16.10	11.2
Tanh	16.56	16.80	7.13	13.16	8.6
ReLU	16.80	11.35	12.30	10.34	9
Softmax	22.78	17.48	18.04	14.85	11.4
Sigmoid	18.68	8.89	8.87	7.92	7.1

## Conclusion

In this work, the security and trustworthiness of the VANET are improved by developing an innovative blockchain-based authentication framework. Here, PUE-FLO algorithm optimizes the parameters of AESNet method to improve accuracy of authentication process. When evaluated against traditional models like DNN, LSTM, GRU, and ESNet, proposed model attained 94% accuracy in authentication process. Compared to RSA, DES and ECC, the proposed model attained a low memory footprint of 6.66% and a reduced decryption time of 30%. The robustness of above 95% is attained by the developed cryptography, and it is used for secure data transfer using the blockchain module.

### Limitations and future scope

In future work, suggest apparoch will be extended and validated using real-time operational datasets collected from practical wireless communication environments to assess its performance under dynamic network conditions, environmental interferences, and heterogeneous traffic patterns. This real-world adoption will further enhance the generalization capability, scalability, and deployment readiness of the framework for practical network security applications. Despite various advantages, this model has some disadvantages too. Firstly, the developed model faces a high computational overhead to implement both cryptography and authentication on a single platform. Then, the latency issues slow the computational rapidity. These limitations paved the way for future directions that will include enhancing privacy-preserving techniques by integrating with other technologies like 5G and edge computing, and will explore decentralized identity management for more secure and efficient vehicular communication. The formal security proof has been done in future work.

## Data Availability

All data generated or analyzed during this study are included in the manuscript.

## Appendix

Critical Success Index (CSI) $Wc_{si}^{d}$ is depicted in Eq. (27).

27 $$Wc_{si}^{d} = \,\frac{{T_{Aa} }}{{T_{Aa} + F_{Ca} + F_{Cb} }}.$$

Mathew Correlation Coefficient (MCC)$WM_{c}^{cf}$ is provided in Eq. (28).

28 $$WM_{c}^{cf} = \frac{{\left( {T_{{^{Aa} }} .T_{{^{Ab} }} } \right) - \left( {F_{{^{Ca} }} .F_{Cb} } \right)}}{{\sqrt {\left( {T_{{^{Aa} }} + F_{{^{Ca} }} } \right)\left( {T_{{^{Aa} }} + F_{Cb} } \right)\left( {T_{{^{Ab} }} + F_{{^{Ca} }} } \right)\left( {T_{{^{Ab} }} + F_{Cb} } \right)} }}.$$

Here, term $T_{Aa}$ is true positive, $T_{Ab}$ is true negative $F_{Ca}$ is false positive, and $F_{Cb}$ is false negative are used.

Balanced Accuracy (BA) $W_{{_{Bal} }}^{Acc}$ in Eq. (29).

29 $$W_{{_{Bal} }}^{Acc} = \left( {\frac{{L_{sn} + L_{sp} }}{2}} \right).$$

Here, $L_{sn}$ is sensitivity and $L_{sp}$ is specificity.

False Omission Rate (FOR)$Lr_{of}$ in Eq. (30).

30 $$Lr_{of} = \frac{{Cb_{v} }}{{Pb_{v} }}.$$

Here, $Cb_{v}$ is false negative value and $Pb_{v}$ is predictive negative value.

Bookmaker Informedness (BM)$L_{{B_{k} M}}$ in Eq. (31).

31 $$L_{{B_{k} M}} =^{Aa} R +^{Ab} R - 1.$$

Here, true positive rate and true negative rate are indicated by $^{Aa} R$ and $^{Ab} R$ , respectively.

Markedness (MK)$L_{MrK}$ in Eq. (32)

32 $$L_{MrK} = Aa_{pv} + Cb_{pv} - 1.$$

Here, true positive predictive value and negative predictive value are indicated by $Aa_{pv}$ and $Cb_{pv}$.

Prevalence Threshold (PT)$L_{PT}$ in Eq. (33).

33 $$L_{PT} = \frac{{\sqrt {^{Aa} R \times^{Ca} R} -^{Ca} R}}{{^{Aa} R \times^{Ca} R}}.$$

Here, positive rate and false positive rate are indicated by $^{Aa} R$ and $^{Ca} R$, respectively.

Fowlkes–Mallows index (FM)$L_{FMI}$ in Eq. (34).

34 $$L_{FMI} = \sqrt {Aa_{pv} \times^{Aa} R} .$$

Here, $Aa_{pv}$ is positive predictive value, and true positive rates are indicated by $^{Aa} R$.

## Abbreviations

BA: Balanced accuracy
BM: Bookmaker informedness
CSI: Critical success index
DTHE-OMK: Dual trapdoor homomorphic encryption with optimal multi-key
F1 Score: F1-score
FM: Fowlkes–Mallows index
FOR: False omission rate
FPR: False positive rate
MCC: Matthew correlation coefficient
MK: Markedness
PT: Prevalence threshold
ROC: Receiver operating characteristic
VANET: Vehicular Ad Hoc network
